# Supplementary material for: Resting heart rate and risk of dementia: a Mendelian randomization study in the international genomics of Alzheimer’s Project and UK Biobank
Source: PeerJ. 2024 Mar 15;12:e17073. doi: 10.7717/peerj.17073 (PMC10946385; doi:10.7717/peerj.17073)
Supplement: Supplemental Information 1 [file peerj-12-17073-s001.docx]

Resting Heart Rate and Risk of Dementia: A Two-Sample Mendelian Randomization Study in the International Genomics of Alzheimer’s Project and UK Biobank

***Supplemental Information***

**Supplemental text**

RHR: Resting heart rate GWAS Summary Statistics

IGAP: Alzheimer Disease GWAS Summary Statistics from the International Genomics of Alzheimer's Project meta-analysis

FA-U and MA-U: Alzheimer Disease GWAS Summary Statistics from the UK Biobank

FH-AD: Alzheimer Disease GWAS meta-analysis of UKBB and IGAP Summary Statistics

**Table S1**. Genome-Wide SNPs associated to Resting heart rate (*P*-value < 5x10^-8^)

**Figure S1**. Sensitivity test by leave-one-SNP-out sensitivity analysis

**Table S2**. Power (two-sided α=0.05) for Two-Sample Mendelian randomization analysis

**Table S3**. Summary of the MR-Based Analysis of Blood Pressure and Dementia using summary statistics from Kunkle et al as outcome dataset (FH-AD 2)

**Figure S2**. Scatter plot of MR analysis using TwoSampleMR R package

**Figure S3**. Forest plot of single SNP of RHR on the risk of AD

**Figure S4**. Scatter plot of bidirectional MR analysis using TwoSampleMR R package

**Table S4**. Summary results of the bidirectional MR Analysis for RHR on AD

**Table S5**. LD-score regression result of the RHR and AD-IGAP summary statistics

**Supplemental References**

**Supplemental Text**

**RHR: Resting heart rate GWAS Summary Statistics**

The hitherto largest genome-wide association study of RHR in up to 265 046 individuals identified 76 loci associated with resting heart rate (*P* < 5 × 10^−8^); 64 of these loci were replicated in 130,795 individuals from 4 cohorts, and 46 loci have not been previously reported as associated with resting heart rate.1 The genome-wide association discovery and replication analysis started with 19.9 million genetic variants to identify loci associated with resting heart rate (*P* < 5 × 10^−8^). The magnitudes of the associations ranged from 0.2 to 1.1 beats per minute (bpm) per effect allele. Collectively, the total variance explained by the 64 loci for resting heart rate was 2.5%. Further information regarding recruitment and diagnostic assessment are reported elsewhere. ^1,2^ Results and data are reported online <https://www.nature.com/articles/ng.3708#citeas>.

**IGAP: Alzheimer Disease GWAS Summary Statistics from the International Genomics of Alzheimer's Project meta-analysis**

The International Genomics of Alzheimer's Project (IGAP)^3^ is a large two-stage study based upon Genome-Wide association studies (GWAS) of AD from individuals of European ancestry aged over 68 for cases and over 65 for controls (age at onset/age at assessment).^3^ In 1-stage meta-analysis, Lambert and colleagues used genotyped and imputed data on 705,5881 SNPs of four previously-published GWAS datasets consisting of 17,008 AD cases and 37,154 controls. The four consortia GWAS datasets were: the European AD Initiative - EADI, the AD Genetics Consortium - ADGC, the Cohorts for Heart and Aging Research in Genomic Epidemiology consortium - CHARGE, and the Genetic and Environmental Risk in AD consortium - GERAD

(<http://web.pasteur-lille.fr/en/recherche/u744/igap/igap_download.php>).

**FA-U and MA-U: Alzheimer Disease GWAS Summary Statistics from the UK Biobank**

UK Biobank data^2^ (http://www.ukbiobank.ac.uk) were collected on over 500,000 individuals aged between 37 and 73 years from across Great Britain (England, Wales and Scotland) at the study baseline (2006 - 2010), including health, cognitive and genetic data. This GWAS related proxy-phenotype information on dementia (i.e., reporting a parent with Alzheimer’s dementia or dementia) to genetic data from 314,278 individuals from the UK Biobank cohort to identify new AD-associated loci. GWA studies were conducted separately for maternal and paternal AD due to a 1.7-fold difference in disease prevalence - 9.6% and 5.5%, respectively.^4^ Data are available in <https://datashare.is.ed.ac.uk/handle/10283/3364>.

**FH-AD: Alzheimer Disease GWAS meta-analysis Summary Statistics**

A recent large 2-stage meta-analysis related proxy-phenotype information on self-reported family history of AD or dementia to genetic data from 314,278 individuals in the UKBB (<http://cnsgenomics.com/data/marioni_et_al_2018_tp/AD_UKB_parents_IGAP_07Feb2019.txt.gz>).^4^ GWAS analyses were conducted separately for maternal (MA-U) and paternal (FA-U) family history and meta-analyzed. In a second stage meta-analysis, summary statistics from the stage 1 meta-analysis were combined with GWAS summary statistics from the IGAP study. Sensitivity analyses showed that an overlap of controls in the maternal and paternal GWAS did not bias the results.

**Genetic Instrument Filtering QC**

A total of 64 SNPs associated with RHR (*P* < 5 × 10^−8^) were selected as candidates. Three of these SNPs (rs35284930, rs11320420, rs11183443) were excluded due to lack of SE and P-value in RHR GWAS. Linkage Disequilibrium between genetic variants was estimated within resting heart rate trait SNP set. Twelve SNPs were excluded from the analysis because of an unresolvable strand ambiguity (RHR GWAS, rs907683, MAF = 0.43; rs13165531, MAF = 0.42; rs3951016, MAF = 0.47; rs58437978, MAF = 0.50; rs41748, MAF = 0.45; rs138186803, MAF = 0.41; rs10739663, MAF = 0.45; rs1994135, MAF = 0.47; rs867400, MAF = 0.43; rs7194801, MAF = 0.43; rs6123471, MAF = 0.46; rs35284930, MAF = 0.42). After searching the PhenoScanner GWAS database,^5^ we removed one SNP (rs12721051) that reached genome-wide significance (*P* < 5 × 10^−8^) in published dementia GWAS (PMID: 24162737). Considering potential confounding, we therefore removed 6 SNPs associated with high blood pressure (HBP) (rs151041685, rs3749237, rs17881696, rs56233017, rs10841486, rs422068). Six SNPs were not available in the IGAP results (rs145358377, rs41317993, rs11454451, rs564190295, rs13002735, rs17180489) and excluded from the related analyses (**Table S1**).

We used GSMR software^6^ to test the presence of SNPs showing evidence of pleiotropic effects by the heterogeneity in dependent instruments outlier analysis (HEIDI-Outlier). None of the SNPs failed the test in the analyses.

**Table S1.** Genome-Wide SNPs associated to Resting Heart Ratio (*P*-value < 5x10^-8^).

| **rsID** | **CHR** | **POS** | **Candidate Genes** | **Effect allele** | **Non-effect allele** | **MAF** | **Exclusions** | **IGAP** | **MA-U** | **FA-U** | **FH-AD** | **FH-AD2^7^** |
| --- | --- | --- | --- | --- | --- | --- | --- | --- | --- | --- | --- | --- |
| rs145358377 | 1 | 6272136 | RNF207nc; ICMTn | GA | G | 0.36 |  | NA | NA | NA | NA | NA |
| rs272564 | 1 | 45012273 | RNF220n | C | A | 0.28 |  |  |  |  |  |  |
| rs2152735 | 1 | 87893132 | LMO4n | A | G | 0.33 |  |  |  |  |  |  |
| rs41317993 | 1 | 207961732 | CD46n; CD34d | A | G | 0.1 |  | NA | NA | NA | NA |  |
| rs11454451 | 1 | 217722890 | GPATCH2n | CT | C | 0.26 |  | NA | NA | NA | NA | NA |
| rs1260326 | 2 | 27730940 | GCKRnc | C | T | 0.39 |  |  |  |  |  |  |
| rs12713404 | 2 | 60006705 | BCL11An | T | G | 0.38 |  |  |  |  |  |  |
| rs564190295 | 2 | 175547672 | WIPF1n | GCCGCCGCCCCC | G | 0.15 |  | NA | NA | NA | NA | NA |
| rs151041685 | 2 | 179725237 | CCDC141ncd; TTNd | T | G | 0.09 | associated with HBP |  |  |  |  |  |
| rs62172372 | 2 | 188242369 | CALCRLne | G | A | 0.19 |  | *P*-value < 0.05 |  |  |  | *P*-value < 0.05 |
| rs907683 | 2 | 220299541 | SPEGnd; DESn | T | G | 0.43 | freq. 0.4-0.6 |  |  |  |  |  |
| rs4608502 | 2 | 228134155 | COL4A3n | C | T | 0.33 |  |  |  |  |  |  |
| rs13002735 | 2 | 232268884 | B3GNT7nc | C | A | 0.24 |  | NA |  |  |  |  |
| rs41312411 | 3 | 38621237 | SCN5And | G | C | 0.15 |  |  |  |  |  |  |
| rs3749237 | 3 | 49770032 | IP6K1n; GMPPBn; FAM212Ad; DAG1d; KLHDC8Bed; LAMB2d; PRKAR2Ad; QRICH1ed | A | G | 0.32 | associated with HBP |  |  |  |  |  |
| rs2358740 | 3 | 53455569 | CACNA1Dn | T | G | 0.32 |  |  |  |  |  |  |
| rs1483890 | 3 | 69410725 | FRMD4Bn | G | A | 0.3 |  |  |  |  |  |  |
| rs11920570 | 3 | 122090102 | CCDC58n | A | G | 0.26 |  |  |  |  |  |  |
| rs7612445 | 3 | 179172979 | GNB4n | T | G | 0.19 |  |  |  |  |  |  |
| rs12501032 | 4 | 23951018 | PPARGC1An | G | C | 0.31 |  |  |  |  |  |  |
| rs6845865 | 4 | 148974602 | ARHGAP10nd; EDNRAd | C | T | 0.16 |  |  |  |  |  |  |
| rs13165531 | 5 | 30888583 | CDH6n | T | A | 0.42 | freq. 0.4-0.6 |  |  |  |  |  |
| rs35284930 | 5 | 137541385 | CDC23n | G | GA | 0.42 | freq. 0.4-0.6/missing SE and *P*-value |  |  |  |  |  |
| rs4868243 | 5 | 172643118 | NKX2-5n | A | G | 0.16 |  |  |  |  |  |  |
| rs236349 | 6 | 36820565 | PPIL1ne | G | A | 0.34 |  |  |  |  |  |  |
| rs3951016 | 6 | 118559658 | SCLC35F1n; PLNd | A | T | 0.47 | freq. 0.4-0.6 |  |  |  |  |  |
| rs1320761 | 6 | 122168138 | GJA1n | T | C | 0.11 |  |  |  |  |  |  |
| rs58437978 | 7 | 35258277 | TBX20n | C | T | 0.5 | freq. 0.4-0.6 |  |  |  |  |  |
| rs180239 | 7 | 93550415 | GNGT1n; GNG11n | C | G | 0.35 |  |  |  |  |  |  |
| rs17881696 | 7 | 100493359 | UFSP1nc; SRRTn; ACHEne; EPHB4d; GIGYF1d; PCOLCEd | A | G | 0.18 | associated with HBP |  |  |  |  |  |
| rs41748 | 7 | 116446573 | METn | G | T | 0.45 | freq. 0.4-0.6 |  |  |  |  |  |
| rs11563648 | 7 | 126970046 | ZNF800n | C | G | 0.27 |  | *P*-value < 0.05 |  |  |  | *P*-value < 0.05 |
| rs138186803 | 7 | 130965408 | MKLN1n | A | AT | 0.41 | freq. 0.4-0.6 |  |  |  |  |  |
| rs73158705 | 7 | 136576100 | CHRM2n | G | A | 0.16 |  |  |  |  |  |  |
| rs56233017 | 8 | 144981488 | PLECn | A | G | 0.04 | associated with HBP |  |  |  |  |  |
| rs10739663 | 9 | 128278739 | MAPKAP1ne | G | A | 0.45 | freq. 0.4-0.6 |  |  |  |  |  |
| rs12576326 | 11 | 44980383 | TP53I11n | G | A | 0.34 |  |  |  |  |  |  |
| rs11320420 | 11 | 61542006 | MYRFn; FEN1e; FADS2e; TMEM258e | G | GA | 0.34 | missing SE and *P*-value |  |  |  |  |  |
| rs75190942 | 11 | 128764571 | KCNJ5nd; C11orf45n | A | C | 0.09 |  |  |  |  | *P*-value < 0.05 |  |
| rs2283274 | 12 | 2184466 | CACNA1Cn | C | G | 0.18 |  |  |  |  |  |  |
| rs10841486 | 12 | 20472202 | PDE3And | C | T | 0.22 | associated with HBP |  |  |  |  |  |
| rs4963772 | 12 | 24758480 | SOX5n | A | G | 0.15 |  |  |  |  |  |  |
| rs1050288 | 12 | 27955296 | KLHL42n | T | C | 0.34 |  |  |  |  |  |  |
| rs1994135 | 12 | 33682405 | SYT10n | C | T | 0.47 | freq. 0.4-0.6 |  |  |  |  |  |
| rs11183443 | 12 | 37945516 | ALG10Bn | C | T | 0.13 | missing SE and *P*-value |  |  |  |  |  |
| rs867400 | 12 | 64976850 | RASSF3nd | C | T | 0.43 | freq. 0.4-0.6 |  |  |  |  |  |
| rs12579753 | 12 | 82219376 | PPFIA2ne | T | C | 0.23 |  |  |  | *P*-value < 0.05 |  |  |
| rs12889267 | 14 | 21542766 | NDRG2n; ARHGEF40ncd; ZNF219d | G | A | 0.16 |  |  |  | *P*-value < 0.05 | *P*-value < 0.05 |  |
| rs422068 | 14 | 23864804 | MYH6nd; MYH7d | C | T | 0.36 | associated with HBP |  |  |  |  |  |
| rs17180489 | 14 | 72885471 | RGS6n | C | G | 0.14 |  | NA |  |  |  |  |
| rs1549118 | 14 | 78379684 | ADCK1n | T | C | 0.28 |  |  |  |  |  |  |
| rs17201923 | 14 | 85796564 | FLRT2n | G | A | 0.28 |  |  |  |  |  |  |
| rs4900069 | 14 | 91583373 | C14orf159n | C | A | 0.37 |  |  |  |  |  |  |
| rs7173389 | 15 | 73663903 | HCN4n; NEO1d | T | A | 0.16 |  |  |  |  |  |  |
| rs3915499 | 16 | 15910743 | MYH11nd | A | G | 0.32 |  |  |  |  |  |  |
| rs7194801 | 16 | 65286870 | CDH11n | C | T | 0.43 | freq. 0.4-0.6 |  |  |  |  |  |
| rs79121763 | 17 | 15195279 | TEKT3n; PMP22d | T | C | 0.09 |  |  |  |  |  |  |
| rs11083258 | 18 | 25766218 | CDH2nd | C | A | 0.17 |  |  |  |  |  |  |
| rs61735998 | 18 | 34289285 | FHOD3ncd | T | G | 0.02 |  |  |  |  |  |  |
| rs16974196 | 19 | 40833470 | C19orf47nd; MAP3K10e | A | G | 0.32 |  |  |  |  |  |  |
| rs12721051 | 19 | 45422160 | APOEn; APOC1n; PVRL2d | G | C | 0.18 | Associated with AD |  |  |  |  |  |
| rs6123471 | 20 | 36840156 | KIAA1755nc | C | T | 0.46 | freq. 0.4-0.6 |  |  |  |  |  |
| rs17265513 | 20 | 39832628 | ZHX3nc; EMILIN3d | C | T | 0.19 |  |  |  |  |  |  |
| rs2076028 | 22 | 39150450 | SUN2n; CBY1e; FAM227Ae; JOSD1e; TOMM22e; DDX17d; GTPBP1d | A | G | 0.29 |  |  | *P*-value < 0.05 |  | *P*-value < 0.05 |  |
|  |  |  |  |  |  |  | Total included SNPs | 35 | 38 | 37 | 36 | 38 |

rsID = SNP Identification number; CHR = chromosome; POS = chromosome position; MAF = minor allele frequency; NA= not available in outcome datasets; FH-AD = Meta-analysis published by Marioni et al 2019; FH-AD 2 = Meta-analysis published by Kunkle et al 2019. IGAP GWAS = SNPs that were associated to AD (*P*-value < 0.05) were excluded. All SNPs were in Hardy-Weinberg equilibrium (HWE) (*P*-value < 1x10^-10^). LD r2 = SNPs with a Linkage Disequilibrium r2>0.001 were excluded.


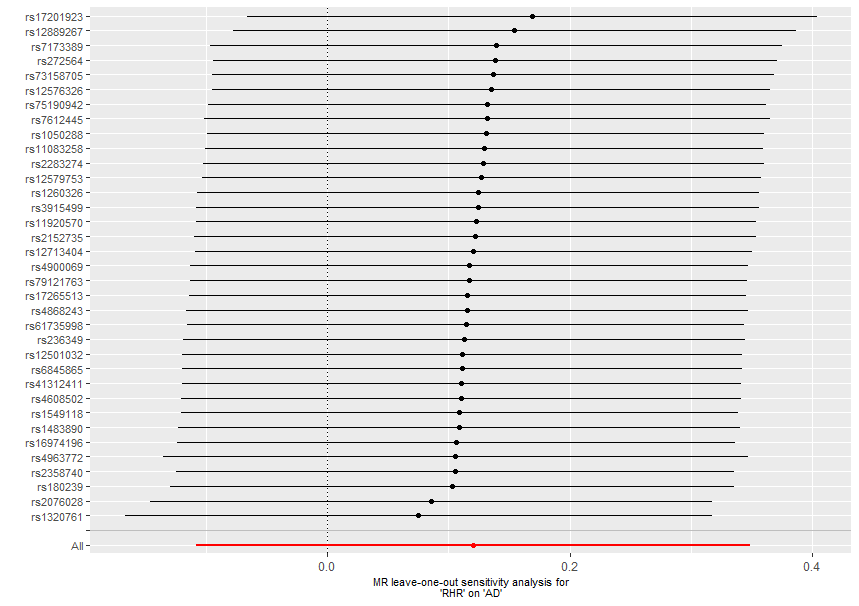


**Figure S1.** Sensitivity test by leave-one-SNP-out sensitivity analysis. RHR is the exposure “Resting Heart Rate”. AD is the outcome “Alzheimer Disease”.

**Table S2.** Power (two-sided α=0.05) for Two-Sample Mendelian randomization analysis

| **Exposure** | **Outcome - AD**  **(Dataset)** | **No.** | **Proportion of cases in the outcome dataset** | **Causal Effect**  **[exp(**βxy**)]** | **F statistics** | **R^2^ of**  **instrument** | **Power**  **(Observed Effect)** | **Power**  **(Theoretical**  **Effect 0.9)** | **Power**  **(Theoretical**  **Effect 0.8)** | **Power**  **(Theoretical**  **Effect 0.7)** |
| --- | --- | --- | --- | --- | --- | --- | --- | --- | --- | --- |
| RHR | IGAP | 54162 | 0.31 | 1.12 | 603.41 | 0.011 | 28.3% | 22.2% | 71.5% | 98.1% |
| RHR | MA-U | 314278 | 0.10 | 0.84 | 3496.51 | 0.011 | 82.8% | 41.9% | 96.1% | 100% |
| RHR | FA-U | 314278 | 0.05 | 0.87 | 3175.53 | 0.011 | 5.6% | 25.2% | 78.2% | 99.2% |
| RHR | FH-AD † | 388324 | 0.19 | 0.97 | 3923.46 | 0.010 | 11.2% | 73.2% | 100% | 100% |
| RHR | FH-AD § | 63926 | 0.34 | 0.89 | 646.72 | 0.011 | 31.1% | 26.3% | 80.3% | 99.4% |

Post-hoc power calculations were based on the method developed by Burgess S.^8^ Causal Effect = exponentiated estimate obtained from Inverse-Variance

Weighted MR (IVW-MR). † (FH-AD) Meta-analysis published by Marioni et al 2019. § (FH-AD 2) Meta-analysis published by Kunkle et al 2019.

A power calculation was also estimated for each analysis using theoretical 10%, 20% and 30% effect sizes (corresponding to 0.9, 0.8 and 0.7 odds ratios).

**Table S3.** Summary of the MR-Based Analysis of RHR and Dementia using summary statistics from Kunkle et al^7^ as outcome dataset (FH-AD 2).

| **Method** | **Resting Heart Rate** | | | | | |
| --- | --- | --- | --- | --- | --- | --- |
|  | **RHR-FH-AD 2** | | | **Adjusting for RHR-modifying medication use** | | |
|  | **No. of SNPs** | **b (SE)** | ***P*-value** | **No. of SNPs** | **b (SE)** | ***P*-value** |
| **GSMR** | 38 | -0.11 (0.10) | 0.29 | 31 | 0.09 (0.11) | 0.39 |
| **IVW (fixed -effects)** | 38 | -0.11 (0.10) | 0.29 | 31 | 0.09 (0.11) | 0.39 |
| **IVW (random-effects)** | 38 | -0.11 (0.09) | 0.24 | 31 | 0.09 (0.08) | 0.35 |
| **MR-Egger** | 38 | -0.25 (0.27) | 0.35 | 31 | 0.13 (0.16) | 0.43 |

The table reports the results for the following MR methods: MR-Egger, Fixed/Random Effect Inverse-Variance-Weighted (IVW) and Generalized-Summary-MR (GSMR) analysis.

In order to compare the results between the GSMR analysis and the other MR analysis the same SNPs were used in each MR method. Beta (b) estimates are reported as standardized Beta values; SE = Standard Error; MR-Egger meta-regression intercept different from zero (*P*-value < .05) was tested as an indication of directional horizontal pleiotropy driving the results. The SNPs used in the RHR analyses are the same as the SNPs used in the IGAP analyses (Table S1).

No horizontal pleiotropy was detected (Pleiotropy *P*-value > .05). A Cochran’s Q heterogeneity *P*-value > .10 was observed in analysis. Analyses used the RHR GWAS summary statistics of SNPs strongly associated to resting heart rate (*P*-value < 5 x 10^-8^) as the exposure. As outcome we used diagnosis of AD (Kunkle et al 1-stage summary statistics; URL: <https://www.niagads.org/igap-rv-summary-stats-kunkle-p-value-data>).


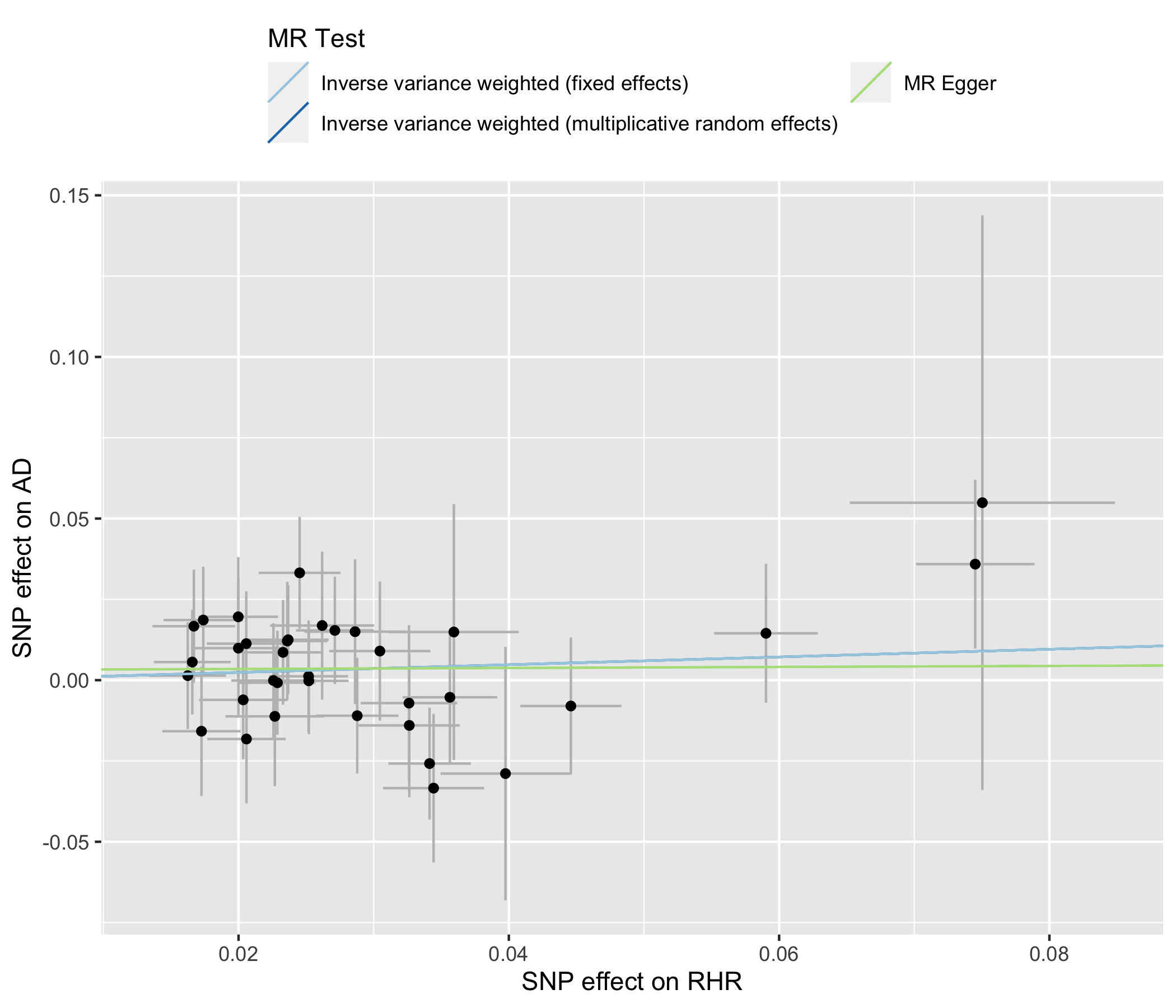


**Figure S2**. Scatter plot of MR analysis using TwoSampleMR R package^9^. Each point on this graph represents a SNP, which the line is the 95% CI for that point. The abscissa indicates the effect of SNP on exposure (RHR), and the ordinate represents the effect of SNP on outcome (AD)


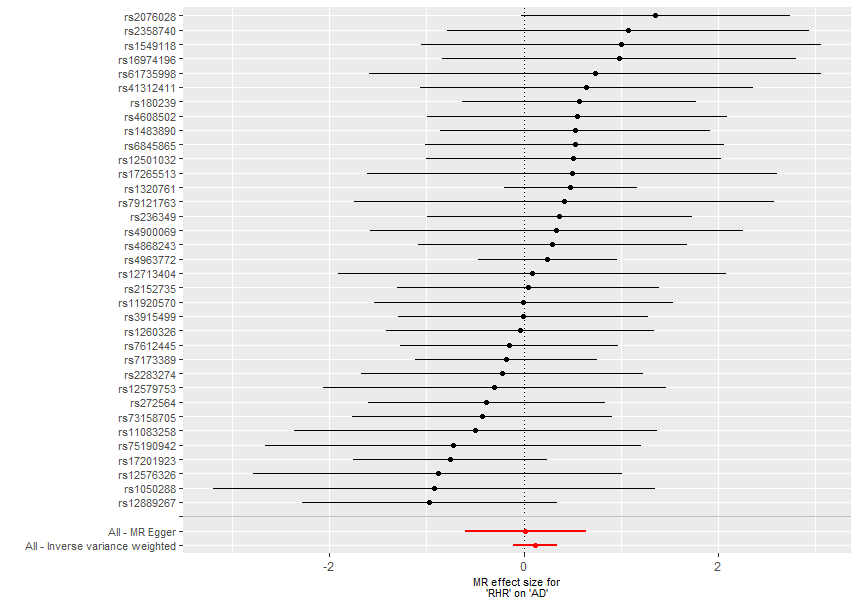


**Figure S3**. Forest plot of single SNP of RHR on the risk of AD. Each horizontal solid line in the plot shows the result estimated by a single SNP using the Wald ratio method. The red line in the bottom represents a combined result using IVW method, which shows the effect of RHR on the risk of AD.


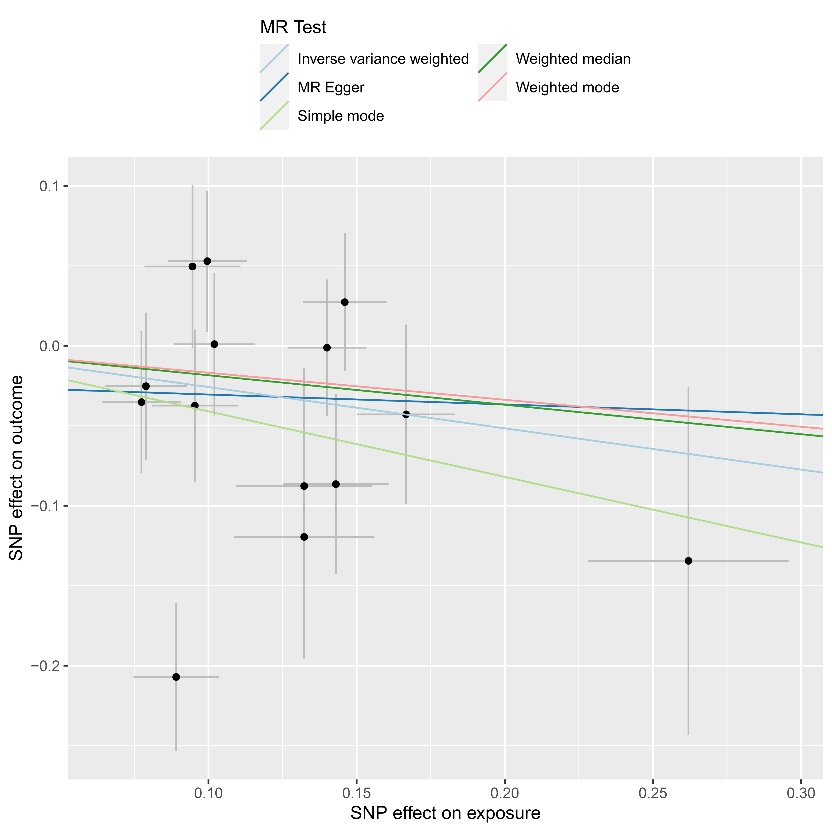


**Figure S4**. Scatter plot of reverse MR analysis using TwoSampleMR R package^9^. Each point on this graph represents a SNP, which the line is the 95% CI for that point. The abscissa indicates the effect of SNP on exposure (AD), and the ordinate represents the effect of SNP on outcome (RHR)

**Table S4**. Summary results of the bidirectional MR Analysis for AD On RHR

| **Methods** | **IGAP AD - RHR dataset** | | |
| --- | --- | --- | --- |
|  | Number of SNPs | β (SE) | *P* |
| IVW (Fixed effects) | 35 | -0.26 (0.17) | 0.120 |
| IVW (Random effects) | 35 | -0.18 (0.16) | 0.264 |
| MR-Egger | 35 | -0.06 (0.61) | 0.921 |

The same SNPs were used in each outcome dataset. In all analyses, there was no horizontal pleiotropy (All MR-Egger Ps > 0.05). The IVW method was additionally used to determine the causal effect.

MR, Mendelian randomization; AD, Alzheimer’s disease; RHR, resting heart rate; IGAP, International Genomics of Alzheimer's Project; IVW, inverse variance weighted; SNP, single nucleotide polymorphism

**Table S5**. LD-score regression^10^ result of the RHR and AD-IGAP summary statistics

| p1 | data/RHR_munged.sumstats.gz |
| --- | --- |
| p2 | **data/AD_munged.sumstats.gz** |
| rg | **0.1309** |
| se | 0.0765 |
| z | 1.7108 |
| p | **0.0871** |
| h2_obs | 0.0328 |
| h2_obs_se | 0.0155 |
| h2_int | 1.0656 |
| h2_int_se | 0.0292 |
| gcov_int | -0.0133 |
| gcov_int_se | 0.0065 |

p1, p2: input files of trait1 and trait2 in LD Score regression genetic correlation computation; rg: genetic correlation estimate; se: standard error of rg; z: z-score for rg; p: P-value for rg; h2_obs, h2_obs_se = observed scale h2 for trait 2 and standard error, h2_int, h2_int_se = single-trait LD Score regression intercept for trait 2 and standard error; gcov_int, gcov_int_se = cross-trait LD Score regression intercept and standard error.

**Supplemental References**

1. Eppinga, R., Hagemeijer, Y., Burgess, S. et al. Identification of genomic loci associated with resting heart rate and shared genetic predictors with all-cause mortality. Nat Genet 48, 1557–1563 (2016).

2. Sudlow, C. et al. UK Biobank: an open access resource for identifying the causes of a wide range of complex diseases of middle and old age. PLoS Med. 12, e1001779 (2015).

3. Lambert JC, Ibrahim-Verbaas CA, Harold D, Naj AC, Sims R, et al. (2013) Meta-analysis of 74,046 individuals identifies 11 new susceptibility loci for alzheimer's disease. Nat Genet 45: 1452-1458.

4. R.E. Marioni, S.E. Harris, Q. Zhang, A.F. McRae, S.P. Hagenaars, W.D. Hill, et al. GWAS on family history of Alzheimer's disease [published correction appears in Transl Psychiatry 2019; 9:161].

5. J.R. Staley, J. Blackshaw, M.A. Kamat, S. Ellis, P. Surendran, B.B. Sun, et al. PhenoScanner: A database of human genotype-phenotype associations Bioinformatics, 32 (2016), pp. 3207-3209.

6. Zhu, Z. et al. (2018) Causal associations between risk factors and common diseases inferred from GWAS summary data. Nat. Commun. 9, 224.

7. Kunkle et al. Genetic meta-analysis of diagnosed Alzheimer’s disease identifies new risk loci and implicates Aβ, Tau, immunity and lipid processing. Nature Genetics, 2019.

8. Burgess S. Sample size and power calculations in Mendelian randomization with a single instrumental variable and a binary outcome. Int J Epidemiol. 2014 Jun; 43(3): 922–929.

9. G. Hemani, J. Zheng, B. Elsworth, K.H. Wade, V. Haberland, D. Baird, et al. The MR-Base platform supports systematic causal inference across the human phenome. Elife, 7 (2018), Article e34408.

10. Bulik-Sullivan, B., Finucane, H., Anttila, V. *et al.* An atlas of genetic correlations across human diseases and traits. *Nat Genet* **47**, 1236–1241 (2015).
